# An approach to integrated management of diabetes in tuberculosis patients: Availability and readiness of the health facilities of Bangladesh

**DOI:** 10.1371/journal.pone.0309372

**Published:** 2024-08-26

**Authors:** Md. Abdur Rafi, Senjuti Semanta, Tasnim Shahriar, Mohammad Jahid Hasan, Md. Golam Hossain

**Affiliations:** 1 Pi Research Consultancy Center, Dhaka, Bangladesh; 2 M Abdur Rahim Medical College, Dianjpur, Bangladesh; 3 Khulna Medical College, Khulna, Bangladesh; 4 Department of Statistics, University of Rajshahi, Rajshahi, Bangladesh; Tokyo Medical and Dental University: Tokyo Ika Shika Daigaku, JAPAN

## Abstract

**Background:**

Comorbidity of diabetes mellitus and tuberculosis (TB) is a major public health concern in low- and middle-income countries including Bangladesh. An integrated approach is required for adequate management of diabetes mellitus and TB. The objective of the present study was to investigate the availability and readiness of the TB care centers of Bangladesh toward diabetic patients’ management.

**Methods:**

The present study was conducted based on existing data obtained from the Bangladesh Health Facility Survey (BHFS) 2017. Data collected from a total of 303 facilities providing TB services were retrieved. The outcome variables of the present study were availability and readiness of the TB health facilities for providing diabetes mellitus service. Readiness was measured for four domains: staff and guidelines, equipment, diagnostic facility and basic medicine. The independent variables were: facility level, management authority and location of the facility. Binary and multiple logistic regression models were constructed for both the outcome variables (availability and readiness) to find out their predictors.

**Results:**

Services for diabetes mellitus were available in 68% of the TB facilities while high readiness was present in 36% of the facilities. For domain-specific readiness index, readiness for the domains of staff and guidelines, equipment, diagnostic facility and basic medicine was reported in 46%, 96%, 38% and 25% facilities respectively. In the logistic regression model, availability of diabetes mellitus services was better in primary level (aOR 2.62, 95% CI 1.78–4.77) and secondary level (aOR 3.26, 95% CI 1.82–9.05) facilities than community facilities. Similarly, readiness of diabetes mellitus care was also better in these facilities (aOR 2.55, 95% CI 1.05–4.71 for primary and aOR 2.75, 95% CI 1.80–4.32 for secondary facilities). Besides, private TB facilities had better availability (aOR 2.84, 95% CI 1.75–5.89) and readiness (aOR 2.52, 95% CI 1.32–4.29) for diabetes mellitus care.

**Conclusion:**

Availability and readiness for providing diabetes mellitus services in TB care providing facilities in Bangladesh is inadequate.

## Introduction

Tuberculosis (TB) is one of the major causes of morbidity and mortality in low- and middle-income countries including Bangladesh, where the estimated incidence and mortality rate for all forms of TB is 221 and 24 per 100000 population respectively [1, 2]. However, the country is experiencing an epidemiological transition in recent decades with an increasing burden of non-communicable diseases. Diabetes mellitus has become a major public health concern in this country nowadays with more than thirteen million affected people [3]. This growing burden of diabetes is contributing to sustained high burden of TB in the community, and the proportion of comorbid diabetes in TB cases is likely to increase over time [4]. In Bangladesh, an estimated of 13% of the TB patients have diabetes and 15.5% have pre-diabetes and more than one-third of these diabetes cases remain undiagnosed [5].

The double burden of TB and diabetes is a serious and growing challenge for the non-resilient health systems of the developing countries like Bangladesh [6, 7]. The National Tuberculosis Control Program (NTP) of the country has shown a substantial increase in rates of case detection and improved treatment outcomes. However, there is still scope of improvements to tackle the challenges of ensuring complete and early case detection, treatment adherence and combating drug resistance [2]. In this context, the comorbidity of diabetes and TB has potential additional implications for these challenges to TB control. Recent evidence showed that diabetes may accelerate the course of TB in co-morbid patents with a higher chance of treatment failure increasing the risk of relapse or death [8–10]. Diabetes may also accelerates the emergence of drug-resistance in TB patients, especially multidrugresistant TB [11]. Moreover, some TB medications may interfere with the glycemic control through drug interactions, and diabetes may interfere with the activity of certain anti-TB drugs [12].

Considering the increasing burden of diabetes and TB comorbidity, a collaborative framework for care and control of TB and diabetes has been proposed by the world health organization (WHO) [13]. This framework suggests that all the patients with TB should be screened and treated for diabetes at the initial phase. It would minimize the burden of diabetes in patients with TB and would result in better treatment outcome. The NTP of Bangladesh has also adopted these recommendations and provides screening, treatment, and follow-up of the TB patients with diabetes to increase access to diabetes services for these patients [14]. In this regard, the readiness of the health facilities designated for TB patient management is therefore essential as it may play a crucial role in the success of the program [15]. There is a paucity of evidence about the readiness of TB care facilities to manage diabetes in our country. Hence, the objective of the present study was to assess the availability and readiness of diabetes management in TB health facilities in Bangladesh.

## Methods

### Study design and data source

The present study was conducted based on a nationally representative cross-sectional survey, the Bangladesh Health Facility Survey (BHFS) 2017 which was a collaborative study implemented by the National Institute of Population Research and Training (NIPORT) and Ministry of Health and Family Welfare (MOHFW) of Bangladesh and the U.S. Agency for International Development (USAID) under the Demographic and Health Survey (DHS) program. The BHFS survey collected information about the availability and readiness of the basic health care services in the primary and secondary health facilities of Bangladesh [16]. Two types of data collection tools were used in the BHFS; Facility Inventory questionnaire, Health Care Provider Interview questionnaire. In the present study, we abstracted data from the Facility Inventory file hence the unit of analysis remained at the facility level.

### Sampling and sample size

**Fig 1:**
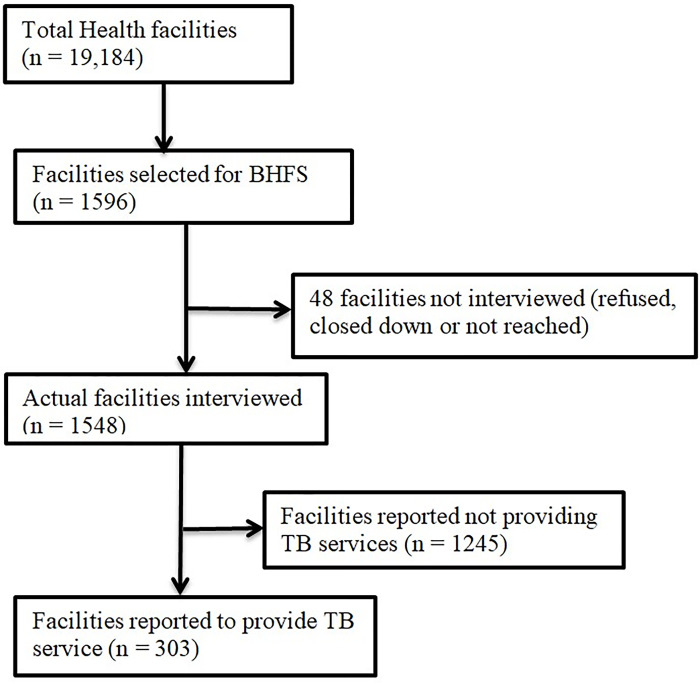
Selection of health facilities included in the current analysis.

A list of total 19,184 registered health facilities were served as the sampling frame for the BHFS which was prepared by NIPORT and MOHFW. A total of 1596 health facilities were selected from all formal-sector health facilities in Bangladesh, both public and private, by stratified random sampling. The public facilities included secondary healthcare facilities such as district hospitals (DHs) and maternal and child welfare centers (MCWCs); primary healthcare facilities such as upazila health complexes (UHCs), upgraded union health and family welfare centers (upgraded UHFWCs), union health and family welfare centers (UHFWCs), union sub-centers/rural dispensaries (USCs/RDs), and community level facilities which were the community clinics (CCs). Community clinics were not considered as a primary level government facility as it has a separate structure based on a public-private partnership. Hence, it was considered as community level facilities. Private facilities included privately run hospitals with at least 20 beds and NGO static clinics/hospitals. These facilities provided outpatient and/or inpatient care for the patients. The allocation of the BHFS sample took the divisional distribution of the health facilities into account as well as other factors such as the precision desired for the results at the national and domain levels and the survey budget. Multi-stage random sampling was used for recruitment of the facilities. Data were successfully collected from 97 percent of the 1596 sampled facilities (n = 1548) [16]. Among these health facilities, 303 facilities that provided tuberculosis services were selected for the present study. The inclusion criteria were the health facility agreeing to participate in the BHFS, being opened during the day of the interview, and providing tuberculosis services which means both diagnosis and treatment of tuberculosis (Fig 1). In the present study, the facilities providing tuberculosis care that included diagnosis and treatment facility for TB were considered for inclusion. Among these facilities, some provided only outpatient-based services like laboratory diagnosis, outpatient based treatment and DOTs service while some other facilities provided inpatient service for TB patients along with outpatient based services.

### Ethical consideration

Formal permission was obtained to use the datasets from the Demographic Health Survey Program accessed at https://dhsprogram.com/data/new-user-registration.cfm.The BHFS 2017 survey was conducted after ethical approval from the Bangladesh Medical Research Council [16]. The informed consent was obtained from the manager, the person-in-charge of the facility, or the most senior health worker responsible for client services who was present at the facility during the survey. All relevant aspects of the study, including its aim and interview procedures were explained clearly to the respondents before interviews. Those respondents agreed their facilities to participate in the study, provided a signed written informed consent. Therefore, the ethical approval for the current study analysis was automatically deemed unnecessary.

### Variables

#### Outcome variables

The first outcome variable of the present study was the “availability” of diabetes services within health facilities providing care for TB. The “availability” in this study was defined as the “percentage of tuberculosis care center offering both diagnostic and treatment facilities for diabetes mellitus”.

The second outcome variable of the present study was the “readiness” of the TB health facilities for providing service for Diabetes patients. The “readiness” in this study was defined as “the capacity of TB care centers to provide management for diabetes mellitus”. This variable was calculated by using an index which comprises four domains as suggested by the WHO-SARA reference manual specific for diabetes services [17]. The first domain of the readiness variable was staff and guidelines which had two indicators: the presence of a guideline for diabetes mellitus management, and at least one staff who received refreshers training in diabetes mellitus diagnosis and treatment within past 24 months. The second domain was equipment which had five indicators: presence of glucometer, blood pressure (BP) measuring machine, weighing scale and stadiometer. The third domain was diagnostics which had three indicators: laboratory facility for blood glucose measurement, urine dipstick for glucose, and urine dipstick for protein. The fourth domain was basic medicine which had four indicators: the availability of the four basic diabetes mellitus medication in the Bangladesh context (metformin, Glibenclamide, insulin injection, and glucose solution). The responses of all the indicators of the domains were aggregated into an index score to calculate a composite score as per the WHO-SARA reference manual [17]. The index score was calculated by adding the presence of each indicator, with equal weight given to each of the domains and each of the indicators within the domains [17, 18]. Since the target was 100%, each domain accounted for 25% (100/4%) of the index. The percentage for each indicator within the domain was equal to 25% divided by the number of indicators within that domain. The overall facility readiness was then calculated as the average of domain indices. The tuberculosis facility that scores at least half (equivalent to the median value of 12.5% and above) in each domain and adding up to the overall of 50% or more were considered to have “high readiness” for the management of TB while those with less than 50% were considered to have “low readiness.” The cut-off point used in this study was also used in the previous studies to dichotomize the outcome variable [18, 19].

#### Independent variables

The independent variables of the present study were: facility level, management authority and location of the facility. The facility type was categorized as ‘community health center’, ‘primary care hospital’ and ‘secondary hospital’; managing authority was categorized as ‘government’ and ‘non-government’, and the location was categorized as ‘urban’ and ‘rural’.

#### Statistical analysis

STATA version 17.0 was used for statistical analyses. All the estimates were weighted in the BHFS 2017 database to correct for non-responses and disproportionate sampling. The results of the current study are presented using descriptive statistics. Binary and multiple logistic regression models were used for both the outcome variables to find association between those and the independent variables. The median odds ratio (MOR) was used to check the existence of clustering effect in outcome variables. The MOR is defined as: $\mathrm{MOR}=\exp\{0.6745\sqrt{2}\sqrt{\sigma_{u}^{2}}\}$ = $\exp(0.95\sqrt{\sigma_{u}^{2}})$, where $\sigma_{u}^{2}$ is the cluster variance, and MOR showed that there was no clustering effect in outcome variables. The magnitude of the standard error (SE) was selected to detect the multicollinearity problem among the independent variables in multiple logistic models, if the SE lies between 0.001 and 0.5, it is judged that there is no evidence of multicollinearity. SE demonstrated that there was no evidence of multicollinearity problem among the independent variables. All the results were interpreted with 95% confidence interval (CI) with an estimated statistical significance level at p<0.05.

## Results

### Baseline characteristics of the facilities

The total number of health facilities reported to provide TB services was 303. Among these facilities, 97 (32%) were community health center, 155 (51%) were primary care hospital and 51 (17%) were secondary hospital. Besides, majority of the facilities were managed by the government (89%). Around half of the facilities (44%) were located in rural areas (Table 1).

**Table 1:** Characteristics of facilities reported to provide TB services (n = 303).

Characteristics	n	%
**Facility level**		
Community health center	97	32.01
Primary care hospital	155	51.16
Secondary hospital	51	16.83
**Management by**		
Government	269	88.78
Private	34	11.22
**Location**		
Rural	134	44.22
Urban	169	55.78

### Availability of diabetes services

Two-thirds of the TB facilities reported providing management of diabetes mellitus (availability of diabetes mellitus services was 68.3%). Services for diabetes mellitus management was available in most of the secondary hospitals (92.2%), privately managed health facilities (91.2%), and urban health facilities (86.4%) (Table 2).

**Table 2:** Availability and readiness for diabetes mellitus management by facility characteristics.

Variables	Total	Facility level			Management authority		Facility location	
		Community health center	Primary care hospital	Secondary hospital	Government	Private	Rural	Urban
Availability (Yes)	68.32	25.77	87.10	92.16	65.43	91.18	45.52	86.39
Readiness (Score >50%)								
Trained personnel	11.55	5.15	16.13	9.80	10.04	23.53	10.45	12.43
Guideline	41.25	14.43	51.61	60.78	39.41	55.88	26.87	52.66
Staff and guidelines domain	45.87	17.53	57.42	64.71	43.49	64.71	31.34	57.40
Glucometer	77.23	94.85	71.61	60.78	76.95	79.41	90.30	66.86
BP machine	84.16	84.54	89.68	66.67	88.10	52.94	88.06	81.07
Weighing scale	91.42	89.69	92.26	92.16	92.19	85.29	90.30	92.31
Stadiometer	41.25	15.46	50.32	62.75	40.15	50.00	24.63	54.44
Equipment domain	95.71	95.88	96.77	92.16	96.28	91.18	95.52	95.86
Blood glucose	58.75	18.56	72.26	94.12	54.28	94.12	27.61	83.43
Urine protein	37.62	17.53	45.16	52.94	31.23	88.24	18.66	52.66
Urine glucose	35.31	15.46	42.58	50.98	29.37	82.35	15.67	50.89
Diagnostics domain	38.28	17.53	45.16	56.86	31.97	88.24	17.16	55.03
Glibenclamide	23.10	3.09	30.32	39.22	21.19	38.24	10.45	33.14
Glucose solution	23.76	3.09	30.32	43.14	20.82	47.06	9.70	34.91
Insulin	17.16	5.15	19.35	33.33	12.27	55.88	5.97	26.04
Metformin	30.36	10.31	32.26	62.75	26.39	61.76	16.42	41.42
Basic medicine domain	25.41	6.19	29.68	49.02	21.19	58.82	9.70	37.87
Overall facility readiness	36.30	11.34	44.52	58.82	31.23	76.47	16.42	52.07

### Readiness of the facilities for diabetes management

High readiness for diabetes mellitus management was present in approximately 36.3% of the included facilities with TB service and the figure was mostly constituted by the secondary hospitals (59%), privately managed facilities (76.5%), and urban facilities (52%) (Table 2). In case of domain-specific readiness index for diabetes mellitus management in TB facilities, almsot 46% facilities reported high readiness in the staff and guidelines domain, 96% facilities reported high readiness in the equipment domain, 38.3% facilities reported high readiness in the diagnostics domain and 25.4% facilities reported high readiness in the basic medicine domain. Detailed domain specific readiness is described in Table 2.

### Predictors of availability and readiness of diabetes mellitus services

In the logistic regression model, the management of diabetes mellitus was more available and ready in higher levels of facilities (aOR 2.62, 95% CI 1.78–4.77 for primary care hospitals and aOR 3.26, 95% CI 1.82–9.05 for secondary hospitals for availability and aOR 2.55, 95% CI 1.05–4.71 for primary care hospitals and aOR 2.75, 95% CI 1.80–4.32 for secondary hospitals for readiness of diabetes mellitus service). Besides, in private TB facilities both availability and readiness for diabetes mellitus care was higher than the government ones (aOR 2.84, 95% CI 1.75–5.89 for availability and aOR 2.52, 95% CI 1.32–4.29 for readiness). No disparity was found between rural and urban facilities in this regard (Table 3).

**Table 3:** Predictors of availability and readiness for diabetes mellitus management in facilities with tuberculosis services (Logistic regression model).

Facility characteristics	Availability		Readiness	
	cOR (95% CI)	aOR (95% CI)	cOR (95% CI)	aOR (95% CI)
Facility level				
Community health center	Ref.	Ref.	Ref.	Ref.
Primary care hospital	1.44 (1.11–3.38)*	2.62 (1.78–4.77) *	6.27 (3.11–12.67) *	2.55 (1.05–4.71) *
Secondary hospital	4.84 (1.07–10.47) *	3.26 (1.82–9.05) *	2.17 (1.82–3.86) *	2.75 (1.80–4.32) *
Management				
Government	Ref.	Ref.	Ref.	Ref.
Private	5.46 (1.63–18.34) *	2.84 (1.75–5.89) *	1.16 (3.11–6.47) *	2.52 (1.32–4.29) *
Location				
Rural	Ref.	Ref.	Ref.	Ref.
Urban	7.60 (4.36–13.24) *	1.40 (0.62–3.16)	5.53 (3.20–9.56) *	1.77 (0.90–3.47)

*p-value <0.05

## Discussion

The major findings of our study were that TB facilities in Bangladesh are still left far behind to manage diabetes mellitus properly. Most of the facilities included in our study were the primary care government facilities which helped us to assess the shortcomings of the root level government facilities. Our study found that diabetes mellitus service was available in approximately two-thirds of the TB service providing health facilities in Bangladesh. Although a small number of private TB facilities were incorporated in our study, diabetes mellitus services were available in majority of those facilities. A previous study also reported that availability of services for diabetes mellitus management is more in private facilities of Bangladesh [20]. The limited availability of equipment, medications, and trained healthcare workers might be the reasons behind the substandard performance of the government facilities [21]. The availability was more in the higher-level facilities than the community health centers which might be contributed by the limited logistic support in these facilities. Studies from other low- and middle-income countries also provided identical results regarding the fewer amenities of the root level health facilities for diabetes mellitus management [22, 23].

On the other hand, the readiness for diabetes mellitus diagnosis and management in the TB health facilities in Bangladesh was poor (around 36%). Moreover, majority of the root level facilities like the community health centers were not even facilitated enough to provide the basic management to the diabetes mellitus patients. These findings are similar to several studies that concluded that most of the health facilities of Bangladesh are not prepared enough for diabetes mellitus management [15, 20]. Similar findings were observed in a previous study from Tanzania where they reported that despite the availability of diabetes mellitus services in around 70% of the TB facilities, only 10% of those were highly ready for providing both diagnostic and treatment to diabetes mellitus patients [18]. Another study from South Africa also illustrated that the bi-directional screening and co-management of TB and diabetes mellitus was weak which is comparable to our study [24]. Although nearly half of the facilities had fulfilled the staff and guidelines domain of the readiness score, hardly any community health centers had trained personnel to manage diabetes mellitus. Moreover, only about one third of the rural facilities had a adequate readiness in this domain which is far behind the urban facilities. Shortage of trained staffs for diabetes mellitus management in rural facilities was also reported by previous studies [15, 25]. Though majority of the TB facilities are well equipped with basic amenities, only half of those were able to carry out blood glucose test for diabetes mellitus diagnosis which is similar to the overall picture of the country [20]. Basic medicine supply was very low in most of the TB facilities, especially in the community health centers and rural facilities. Only a few community health centers (6%) and rural facilities (10%) had met the target level. Metformin was the highest available drug in these facilities. Some previous studies from Bangladesh also revealed that the basic medicine domain was the most deprived sector which indicates that there is a massive shortage of adequate medicine supply for the management of diabetes mellitus [15, 20].

The integration of diabetes screening and treatment within tuberculosis (TB) care, as proposed by the World Health Organization (WHO) has been adopted by the National Tuberculosis Program (NTP) of Bangladesh. Despite the fact, we found that the healthcare facilities of the country are lagged behind to provide the integrated care for TB and diabetes mellitus as majority of the facilities lack availability and readiness of diabetes care. For successful implementation of the integrated service policy for TB and diabetes mellitus, adequate availability of the trained staff, equipment, diagnostic and management facilities would be increased in all level of health facilities.

### Strengths and limitations

Several studies have investigated the availability and readiness of diabetes mellitus services in the health facilities in Bangladesh. However, there is hardly any study which investigated the availability and readiness of the integrated service for TB and diabetes mellitus in this country. The present nationally representative study provides an overview of this issue. It would be the initial step to establish an integrated management system for both TB and diabetes in a high burden setting like Bangladesh. However, the study has some limitations. Firstly, the study only investigated for the very basic facilities for diabetes management. This study did not assess the availability of other important diabetes mellitus diagnostic methods such as glycosylated hemoglobin (HbA1c) assay. Data from tertiary care facilities was not available in the BHFS survey. Further studies are required for prioritizing the requirements and ensuring the services which can build a sustainable infrastructure to implement the collaborative management goal of TB and diabetes mellitus in Bangladesh.

## Conclusions

The findings of the study indicate that the majority of the TB care providing facilities in Bangladesh are not ready enough to provide service for diabetes It is necessary to initiate the refinement of the infrastructures to implement the collaborative framework for the care and control of both TB and diabetes mellitus in this high-burden country. This study can be the first evidence-based information for the policymakers and stakeholders of Bangladesh to take timely initiatives.

## Abbreviations

TB: tuberculosis
BHFS: Bangladesh Health Facility Survey
CI: Confidence Interval
aOR: Adjusted odds ratio
NTP: National Tuberculosis Control Program
WHO: World health organization
NIPORT: National Institute of Population Research and Training
MOHFW: Ministry of Health and Family Welfare
USAID: U.S. Agency for International Development
MCWCs: Maternal and child welfare centers
CCs: community clinics
MOR: Median odds ratio

## References

[1] Zumla A, George A, Sharma V, Herbert RHN, Oxley A, Oliver M. The WHO 2014 global tuberculosis report—further to go. The Lancet Global Health. 2015;3: e10–e12. doi: 10.1016/S2214-109X(14)70361-4 25539957

[2] Kuddus MA, Meehan MT, Sayem M, McBryde ES, et al. Scenario analysis for programmatic tuberculosis control in bangladesh: A mathematical modelling study. Scientific reports. 2021;11: 1–17. 33623132 10.1038/s41598-021-83768-y PMC7902856

[3] Sun H, Saeedi P, Karuranga S, Pinkepank M, Ogurtsova K, Duncan BB, et al. IDF diabetes atlas: Global, regional and country-level diabetes prevalence estimates for 2021 and projections for 2045. Diabetes research and clinical practice. 2022;183: 109119. doi: 10.1016/j.diabres.2021.109119 34879977 PMC11057359

[4] Zheng C, Hu M, Gao F. Diabetes and pulmonary tuberculosis: A global overview with special focus on the situation in asian countries with high TB-DM burden. Global health action. 2017;10: 1264702. doi: 10.1080/16549716.2016.1264702 28245710 PMC5328328

[5] Sarker M, Barua M, Guerra F, Saha A, Aftab A, Latif AM, et al. Double trouble: Prevalence and factors associated with tuberculosis and diabetes comorbidity in bangladesh. PloS one. 2016;11: e0165396. doi: 10.1371/journal.pone.0165396 27798659 PMC5087880

[6] WHO. Integrated care for tuberculosis (TB) and diabetes mellitus(DM) comorbidity in Asian countries: Health system challenges and opportunities. 2022.

[7] Fazaludeen Koya S, Lordson J, Khan S, Kumar B, Grace C, Nayar KR, et al. Tuberculosis and diabetes in india: Stakeholder perspectives on health system challenges and opportunities for integrated care. Journal of Epidemiology and Global Health. 2022;12: 104–112. doi: 10.1007/s44197-021-00025-1 35006580 PMC8907360

[8] Al-Rifai RH, Pearson F, Critchley JA, Abu-Raddad LJ. Association between DM and active tuberculosis: A systematic review and meta-analysis. PloS one. 2017;12: e0187967. 29161276 10.1371/journal.pone.0187967 PMC5697825

[9] Gautam S, Shrestha N, Mahato S, Nguyen T, Mishra SR, Berg-Beckhoff G. Diabetes among tuberculosis patients and its impact on tuberculosis treatment in south asia: A systematic review and meta-analysis. Scientific reports. 2021;11: 1–12. 33483542 10.1038/s41598-021-81057-2 PMC7822911

[10] Huangfu P, Ugarte-Gil C, Golub J, Pearson F, Critchley J. The effects of diabetes on tuberculosis treatment outcomes: An updated systematic review and meta-analysis. International Journal of Tuberculosis and Lung Disease. 2019;23: 783–796. doi: 10.5588/ijtld.18.0433 31439109

[11] Tegegne BS, Mengesha MM, Teferra AA, Awoke MA, Habtewold TD. Association between DM and multi-drug-resistant tuberculosis: Evidence from a systematic review and meta-analysis. Systematic reviews. 2018;7: 1–13. 30322409 10.1186/s13643-018-0828-0 PMC6190557

[12] Nijland HM, Ruslami R, Stalenhoef JE, Nelwan EJ, Alisjahbana B, Nelwan RH, et al. Exposure to rifampicin is strongly reduced in patients with tuberculosis and type 2 diabetes. Clinical Infectious Diseases. 2006;43: 848–854. doi: 10.1086/507543 16941365

[13] WHO. Collaborative framework for care and control of tuberculosis and diabetes. World Health Organization; 2011. 26290931

[14] Hossain MD, Ahmed JU, Rahim MA, Musa A, Latif ZA. Bangladesh national guidelines on the management of tuberculosis and DM co-morbidity (summary). Indian Journal of Endocrinology and Metabolism. 2016;20: 853. 27867891 10.4103/2230-8210.192898 PMC5105572

[15] Biswas T, Haider MM, Gupta RD, Uddin J. Assessing the readiness of health facilities for diabetes and cardiovascular services in bangladesh: A cross-sectional survey. BMJ open. 2018;8: e022817. doi: 10.1136/bmjopen-2018-022817 30385441 PMC6252707

[16] Bangladesh health facility survey 2017: National institute of population research and training, ministry of health and family welfare, dhaka, bangladesh. 2018.

[17] WHO. Service availability and readiness assessment (SARA).

[18] Shayo FK, Shayo SC. Readiness of healthcare facilities with tuberculosis services to manage diabetes mellitus in Tanzania: A nationwide analysis for evidence-informed policy-making in high burden settings. PloS one. 2021;16: e0254349. doi: 10.1371/journal.pone.0254349 34252144 PMC8274870

[19] Shayo FK, Shayo SC. Availability and readiness of diabetes health facilities to manage tuberculosis in tanzania: A path towards integrating tuberculosis-diabetes services in a high burden setting? BMC public health. 2019;19: 1–7. 31412829 10.1186/s12889-019-7441-6 PMC6692934

[20] Chowdhury HA, Paromita P, Mayaboti CA, Rakhshanda S, Rahman FN, Abedin M, et al. Assessing service availability and readiness of healthcare facilities to manage diabetes mellitus in bangladesh: Findings from a nationwide survey. PloS one. 2022;17: e0263259. doi: 10.1371/journal.pone.0263259 35171912 PMC8849622

[21] Basu S, Andrews J, Kishore S, Panjabi R, Stuckler D. Comparative performance of private and public healthcare systems in low-and middle-income countries: A systematic review. PLoS medicine. 2012;9: e1001244. doi: 10.1371/journal.pmed.1001244 22723748 PMC3378609

[22] Rogers HE, Akiteng AR, Mutungi G, Ettinger AS, Schwartz JI. Capacity of ugandan public sector health facilities to prevent and control non-communicable diseases: An assessment based upon WHO-PEN standards. BMC health services research. 2018;18: 1–13. 30081898 10.1186/s12913-018-3426-x PMC6080524

[23] Simão CCAL, Costa MB Colugnati FAB, Paula EA de, Vanelli CP, de Paula RB Quality of care of patients with diabetes in primary health services in southeast brazil. Journal of environmental and public health. 2017;2017. doi: 10.1155/2017/1709807 29129980 PMC5654281

[24] Almossawi H, Matji R, Pillay Y, Singh S, Mvusi L, Mbambo B, et al. Primary health care system readiness for diabetes mellitus and tuberculosis service integration in south africa. J Trop Dis. 2019;7: 329.

[25] Rawal LB, Kanda K, Biswas T, Tanim MI, Poudel P, Renzaho AM, et al. Non-communicable disease (NCD) corners in public sector health facilities in bangladesh: A qualitative study assessing challenges and opportunities for improving NCD services at the primary healthcare level. BMJ open. 2019;9: e029562. doi: 10.1136/bmjopen-2019-029562 31594874 PMC6797278

